# Evaluation of Clinical and Radiological Outcomes in the Management of Intracapsular Fracture Neck of Femur in Elderly Treated With Cemented Bipolar Prosthesis

**DOI:** 10.7759/cureus.55283

**Published:** 2024-02-29

**Authors:** Ajay Lucas, Afwaan Faizal

**Affiliations:** 1 Radiodiagnosis, Saveetha Institute of Medical and Technical Sciences (SIMATS) Saveetha Medical College and Hospital, Chennai, IND

**Keywords:** fracture femoral neck, radiology, elderly patients, intracapsular femoral neck fractures, cemented bipolar prosthesis

## Abstract

Introduction: Hip fractures, including femoral neck fractures (FNFs), represent a significant health challenge globally. Fractures of the hip can be categorized as either intracapsular or extracapsular. Among the elderly, FNFs are particularly prevalent and account for approximately half of all hip fractures.

Aim: This study aimed to evaluate the clinical and radiological outcomes of intracapsular FNFs in the elderly treated with cemented bipolar prostheses.

Objectives: This study aims to: (i) assess the clinical outcomes, including pain relief, functional mobility, and patient satisfaction, in elderly individuals with intracapsular FNFs treated with cemented bipolar prosthesis; (ii) examine the radiological outcomes of intracapsular FNFs in the elderly following treatment with cemented bipolar prosthesis, focusing on factors such as implant stability, fracture healing, and any signs of complications.

Methods: A prospective study included elderly patients (aged 55 and above) with intracapsular FNFs treated with cemented bipolar prostheses. Data were collected using a structured proforma, and outcomes were assessed through clinical and radiological evaluations at regular follow-ups.

Results: The study included 60 participants with a mean age of 65.25 years. Most fractures were subcapital, and the majority of participants did not experience complications after surgery. The average length of the pre-operative hospital stay was 2.35 days, and the post-operative hospital stay was 6.75 days. Functional outcomes, evaluated using the Harris Hip Scoring System, showed varying degrees, with 70% of participants experiencing good outcomes.

Conclusion: The management of intracapsular femoral neck fractures in the elderly with cemented bipolar prostheses demonstrated favorable outcomes, including low morbidity, simple operative procedures, and satisfactory early functional results. The study supports the recommendation of cemented bipolar prostheses for femoral neck fractures in individuals over 60, emphasizing their superiority over bipolar hemiarthroplasty. The results contribute valuable insights for treatment decisions in hip fractures, especially considering evolving reimbursement mechanisms and merit-based incentive payments.

## Introduction

Femoral neck fractures (FNFs) are a subset of proximal femur fractures. The term "hip fractures" is commonly used to describe fractures in the proximal portion of the femur, encompassing the femoral neck, trochanteric region, or subtrochanteric section [1]. While the acetabulum and femoral head are integral to the hip, the term is primarily associated with fractures in specific femoral regions. The global impact of hip fractures is substantial, both in terms of individual health and economic burden. Despite challenges in accurately quantifying global occurrences, estimates from 2000 suggest approximately 1.6 million cases, with projections indicating a rising trend [2]. The majority of hip fractures fall into the categories of intracapsular (cervical) or extracapsular (trochanteric) [3], each with distinct characteristics and treatment approaches.

FNFs constitute about 50% of all hip fractures, particularly affecting the elderly population with chronic osteoporosis. The incidence increases with age, and women in menopause face a higher risk [4]. The epidemiology varies globally, with higher rates in predominantly white communities in certain regions. The occurrence rates, however, have been rising globally, and projections indicate a continued increase [5], especially in countries with aging populations. The classification of FNFs is crucial for understanding their nature and guiding appropriate treatment. Various systems, including the Garden classification, help categorize FNFs based on displacement degree. Displaced intracapsular FNFs often require hip reconstruction techniques [6], involving procedures like hip hemiarthroplasty or total hip arthroplasty (THA).

The pathophysiology of FNFs involves breaks at the proximal end of the femur, often related to factors including serious injury such as falls or underlying bone weakness, such as in osteoporosis. Understanding the anatomical implications is crucial for selecting appropriate treatment options. Cannulated screw fastening is often recommended for elderly patients with nondisplaced, intracapsular FNFs [3], while younger patients with displaced fractures may require alternative stabilization systems. Complications associated with FNFs, especially in elderly patients, include prosthetic joint infections (PJIs), extended antibiotic therapy, revision surgeries, and prolonged hospital stays. PJIs, in particular, necessitate debridement, antibiotics, and implant preservation for treatment. The choice of surgical care depends on factors such as age, bone strength, and fracture type, with increased mortality rates observed in hip fracture patients. [7].

Accurate diagnosis of hip fractures, including FNFs, is crucial for effective management. Radiographs, particularly in two planes, remain a standard diagnostic tool, with additional imaging modalities such as magnetic resonance imaging proving valuable in certain cases [8]. The classification of hip fractures into intracapsular and extracapsular, along with further sub-classifications, aids in tailoring treatment approaches [9]. The commonly used Garden classification helps assess anteroposterior displacement in FNFs, with various other classification systems providing additional insights. Intertrochanteric and subtrochanteric fractures are also considered, with differing implications for blood flow and healing processes. These classifications guide the selection of appropriate surgical approaches and interventions. [10].

Treatment options for hip fractures, including FNFs, encompass both nonsurgical and surgical approaches. Nonoperative procedures may be considered in vulnerable or moribund patients, although they are associated with increased risks such as further fracture displacement and mortality [11]. Internal fixation (IF) remains a common nonsurgical option for non-displaced FNFs, involving closed reduction and screw stabilization. Surgical treatments vary based on factors such as age, fracture type, and patient demand. For displaced FNFs, hip reconstruction techniques, such as hip hemiarthroplasty or THA, are often recommended. The choice between THA and hemiarthroplasty depends on factors like age and displacement degree, with THA associated with increased risks of complications such as dislocation and infection [12]. Prosthetic designs and surgical approaches continue to evolve to improve patient outcomes and reduce complications. Intracapsular fractures, particularly FNFs, may require complete hip arthroplasty for effective treatment. The use of prostheses and fixation methods, such as cannulated screws, depends on factors like displacement and patient characteristics. Complications associated with surgical interventions, including dislocation and infection [13], necessitate careful consideration of treatment approaches.

FNFs in elderly patients present unique challenges in terms of surgical care. Physically demanding procedures, including hip hemiarthroplasty and THA, are complicated by severe inflammation, dislocation, and intraoperative fractures. Prosthetic designs and surgical approaches must be carefully selected to minimize complications associated with extended immobilization, especially in elderly patients [14]. Managing displaced FNFs in the elderly population is a critical aspect of treatment. Hemiarthroplasty is a common procedure involving resection of the femoral head and neck, with variations such as unipolar or bipolar heads. The choice of prosthetic heads influences outcomes, with some studies indicating higher dislocation rates with bipolar heads compared to unipolar heads [15]. These considerations highlight the importance of tailored treatment strategies for elderly patients with FNFs.

This study aimed to evaluate the clinical and radiological outcomes of intracapsular femoral neck fractures in the elderly treated with cemented bipolar prostheses.

## Materials and methods

The study included all patients aged 55 and above. The prospective study spanned from September 2018 to March 2020, with a focus on patients meeting inclusion criteria and excluding those below 55 years with femoral head fractures, muscular dystrophies, mental illnesses affecting post-operative cooperation, and pathological fractures.

A minimum of 60 cases were examined, with a follow-up period of two years. The comprehensive evaluation during follow-ups involved clinical and radiological assessments, considering factors such as limb length discrepancies, post-surgery movements, complications, and the time required for union. The data collection process utilized a structured proforma, with information recorded upon admission, regular observations during hospital stays, and outpatient department follow-ups. Those not attending scheduled visits were contacted via phone, and their follow-up details were documented.

The surgical procedure involved a preoperative surgical profile and spinal anesthesia. The technique emphasized precise prosthesis fitting to prevent gaps, ensuring stable fixation. The study employed a varied approach, including dissection, fascial plane development, cauterization, and capsule release. The surgical steps included internal rotation, dislocation, head resection, canal preparation, trial implantation, cementation, and final implant placement. Postoperatively, patients received intravenous antibiotics and analgesics, with suture removal on the 10th postoperative day.

Preoperative management included detailed history-taking, clinical assessments, Buck's traction application, and necessary investigations. Therapeutic exercises were taught preoperatively, and patients were educated about the surgery and associated risks, obtaining written consent. Intravenous antibiotics and tetanus immunizations were administered one hour before surgery. 

In the postoperative phase, patients who underwent spinal anesthesia received foot end elevation based on their postoperative blood pressure. The initial 24 hours involved meticulous monitoring of blood pressure, pulse rate, temperature, and respiratory rate every half an hour. Blood transfusions were administered as needed, and intramuscular analgesics were provided based on patient compliance, accompanied by a five-day course of intravenous antibiotics. Both lower limbs were maintained in an abducted position with a pillow between them, and drain removal took place after 48 hours. A radiograph was taken at this point, and patients gradually progressed to sitting straight on the second day, standing with support on the third day, and fully weight-bearing with a walker on the fourth postoperative day, depending on pain tolerance. Patients were discouraged from sitting cross-legged or squatting. Suture removal occurred on the tenth postoperative day, and patients were assessed for shortening or deformities before discharge. Those with infections and bedsores received appropriate treatment before discharge.

Subsequent follow-ups occurred at six months, 12 months, 18 months, and 24 months, with functional outcomes analyzed using a modified Harris Hip Scoring System. Radiographs were taken at each follow-up to assess the hip's radiological status. Patients were scheduled for follow-ups at discharge and encouraged to return for further assessments. Clinical examinations, according to the Harris Hip Scoring System, evaluated aspects such as pain, limp, use of support, walking distance, ability to climb stairs, put on shoes and socks, sit on a chair, enter public transportation, leg length discrepancy, and movements. The Harris Hip Scoring System classified total functional outcomes as poor (score < 70), fair (score 71-80), good (score 81-90), and excellent (score 91-100).

Statistical analysis involved calculating the mean of the postoperative Harris Hip Scoring System scores for patients, utilizing the frequency procedure to provide descriptive statistics and graphical displays for various variables.

## Results

Sixty patients in all who had visited the Department of Orthopaedics made up the study group. The study participants' baseline characteristics are displayed in Table 1. 39 patients (65% of the population) were males, and 21 patients (35%) were females in our study. The participants in the study had an average age of 65.25±6.34 years. Thirty (50%) occurrences of left- and right-sided fractures were found among the study subjects. Our analysis found that there were somewhat more cases of the slip and fall category (n = 28; 55%) than road traffic accident cases (n = 27; 45%). The study participants' injury mechanisms are defined by both case categories. A maximum of 36 study participants (or 60%) did not sustain any injuries as a result of a slip and fall or a traffic accident in the current study.

**Table 1:** Baseline characteristics of the study participants

Parameter	Total number of participants: n=60 (%)
Gender	
Male	39 (65)
Female	21 (35)
Laterality of fracture	
Right	30 (50)
Left	30 (50)
Mechanism of injury	
Slip or fall	33 (55)
Road traffic accident	27 (45)
Associated injury	
Abrasions	15 (25)
Head injury	6 (10)
Vertebral compression fracture	3 (5)
None	36 (60)
Co-morbidities	
Diabetes mellitus	15 (25)
Hypertension	6 (10)
Diabetes mellitus + hypertension	9 (15)
None	30 (50)
Type of fracture	
Sub-capital	45 (75)
Transcervical	15 (25)

The average duration of hospital stay before surgery for the research subjects was 2.35 days. The average duration of hospital stay following surgery for the research subjects was 6.75 days. All of the research subjects were treated using a cemented bipolar implant. However, the majority of study participants (70%) did not encounter any difficulties following surgery, even after utilizing a cemented bipolar prosthesis. There were only two cases of periprosthetic fractures among the study participants. 

**Figure 1:**
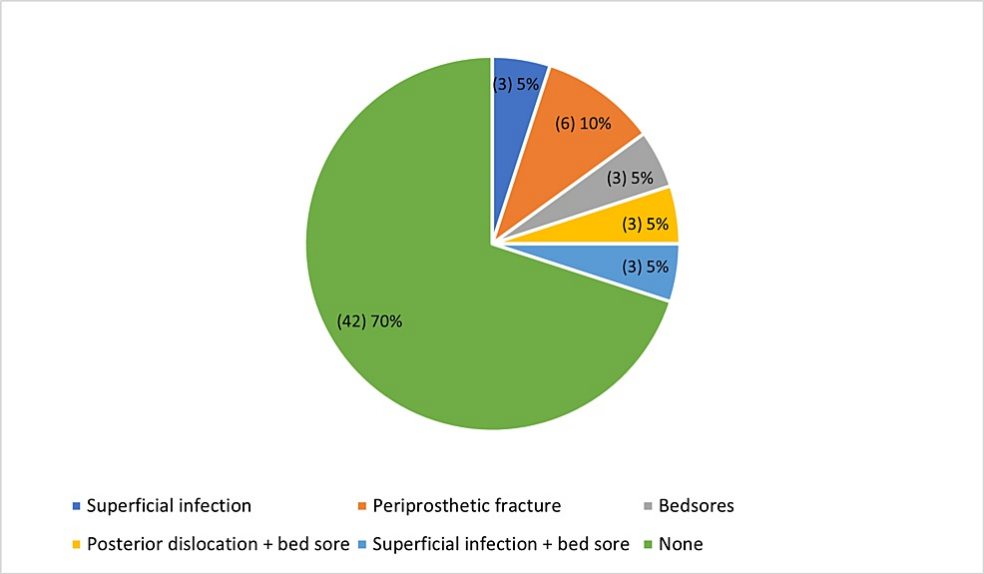
Complications of index procedure in the study participants

However, as Figure 1 illustrates, just one instance of each of the following conditions was noted: superficial infection, bedsores, posterior dislocation with bed soreness, and superficial infection with bed soreness.

**Figure 2:**
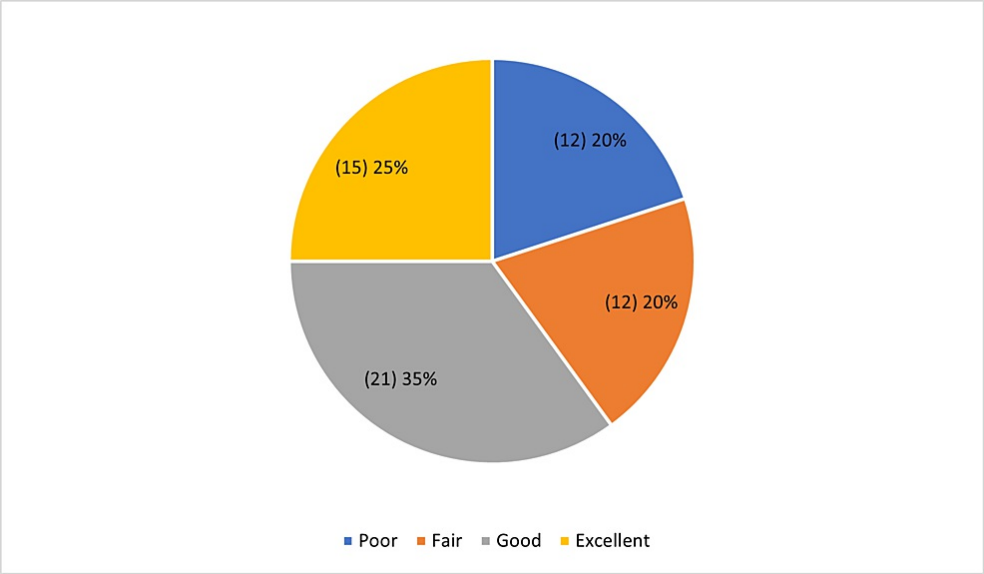
postoperative functional outcome of the study participants using Harris Hip Scoring System

The postoperative functional outcome of the study participants showed that 20% (12) of the patients showed a poor outcome, and 20% (12) of them showed a fair outcome. 35% (21) of the study participants showed good outcomes, followed by excellent outcomes, which are 25% (15), as shown in Figure 2.

**Figure 3:**
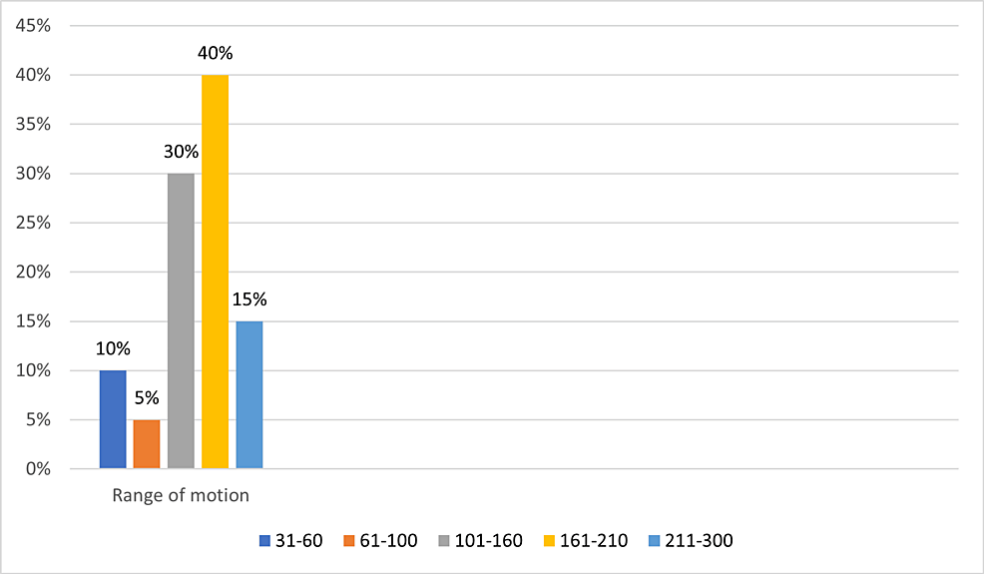
Range of motion in the study participants

Twenty-four (40%) of the study participants had the maximum range of motion (161-210) measured. The study participant with the least range of motion (61-100) was 3 (5%), as shown in Figure 3.

**Figure 4:**
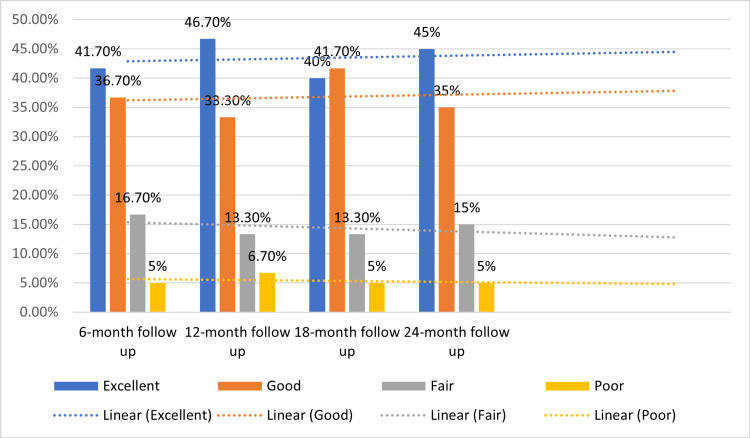
Harris Hip scoring in the study participants during follow-up

Over the course of the follow-up period, functional outcomes were assessed using the modified Harris Hip Scoring System. At the six-month follow-up, the majority of patients exhibited favorable outcomes, with 41.7% scoring in the Excellent range (91-100), followed by 36.7% in the Good range (81-90). A smaller proportion of patients fell into the Fair category (16.7%), and 5% of patients scored in the Poor range (<71). Progressing to the 12-month follow-up, the distribution of scores shifted slightly, with 46.7% achieving an Excellent score, 33.3% falling into the Good category, 13.3% in the Fair range, and a minor increase to 6.7% in the Poor range. At the 18-month follow-up, the Excellent and Good categories remained prominent, encompassing 40% and 41.7% of patients, respectively. The Fair and Poor categories exhibited similar patterns to the 12-month follow-up. Finally, at the 24-month follow-up, the trend of positive outcomes persisted, with 45% achieving an Excellent score and 35% falling into the Good range. The distribution in the Fair and Poor categories remained relatively stable across the follow-up period. These results suggest an overall positive trajectory in functional outcomes, with a majority of patients experiencing either Excellent or Good scores throughout the 24-month follow-up, as shown in Figure 4.

Table 2 demonstrates that half of the research participants reported having moderately severe discomfort. For 18 (30%) of the research participants, this was followed by minor pain. In the current investigation, six patients each reported experiencing severe discomfort and pain while sleeping. Half of the individuals in the current study reported not having a limp. However, only 12 (20%) of the study's participants reported having a minor limp. In the current investigation, three patients reported mild, moderate, and severe limps. Half of the participants in this study did not use a cane at all. Nine (15%) research participants then reported using a cane to walk long distances. In the current study, both subjects could not walk and relied on one crutch. One research subject carried two crutches in addition to a cane for extended walks. Following surgery, about 54 (or 90%) of the study participants are now walking. After the procedure, only the remaining six (10%) are still unable to walk.

**Table 2:** Post-procedure complications in the study participants

Parameter	Total number of participants: n=60 (%)
Pain	
Mild	18 (30)
Moderate	30 (50)
Severe	6 (10)
Pain at bed	6 (10)
Limp	
Slight	12 (20)
Moderate	9 (15)
Severe	9 (15)
No limp	30 (50)
Cane usage	
No	30 (50)
For long distance	12 (20)
Most of the time	3 (5)
One crutch	6 (10)
Two crutches	3 (5)
Unable to walk	6 (10)

## Discussion

Femoral neck fractures remain a perplexing mystery for orthopedic surgeons. Treatment outcomes have shown variations, including osteosynthesis, hemireplacement, and complete hip replacement. Osteosynthesis is less favorable for the elderly due to potential secondary treatment and uncertain results from a second surgery, leading surgeons to commonly opt for prosthetic replacement. In a developing country like India, THA is often not the primary choice due to its physical demands and cost, making hemi-replacement a preferred alternative.

With this rationale, our study aimed to investigate the visual and radiological outcomes of a cemented bipolar prosthesis in patients undergoing treatment for sub-capital and transcervical fractures. Subcapital femoral neck fractures often affect older age groups. Our findings align with other research indicating an increase in subcapital fractures in men, particularly with age, possibly linked to greater trabecular bone loss. Despite the prevalent belief that hip fractures correlate with osteoporosis, evidence suggests a complex relationship, especially in women. The study's sample group, though relatively older, presented with hip fractures possibly correlated with osteoporosis and severe osteoporosis, as indicated by the presence of spinal fractures. Although it is believed that femoral neck fractures are mostly due to osteoporosis, they can be multifactorial. The study observed a higher incidence of fractures in males across age groups, potentially linked to the increasing male-to-female ratio and lower bone density in men. Comparing the sex distribution in our study (65% males, 35% females) with another study that reported a similar sex bias in hip fractures reinforces the consistent observation that males are more predisposed to this injury. This reinforces the need for gender-specific considerations in preventative measures and treatment strategies [7]. Notably, accidents such as tripping or slipping were common causes of fractures, especially in the elderly, emphasizing the importance of preventive care and early medical intervention. Additionally, the study highlighted the association between fractures and road traffic accidents, with a considerable number of cases in the 61-70-year-old age group. Our study's predominant mechanism of injury slips and falls (55%) is in line with the findings of a study where 60% of hip fractures resulted from falls [10]. Both studies underscore the significance of preventive measures targeting fall-related injuries, especially in the elderly population.

Diabetes mellitus emerged as a significant factor in elevating the risk of fractures, even with an average or elevated bone mineral density. Trabecular bone degradation, exacerbated by diabetes, underscores the role of bone quality in skeletal fragility. Postmenopausal osteoporosis in women, attributed to estrogen deficiency, results in accelerated bone resorption and increased fracture risk.

The historical progression of cemented hip arthroplasty was outlined, emphasizing the evolution of surgical techniques to enhance stability and reduce complications. Nevertheless, concerns were raised about potential risks associated with cemented prostheses, such as periprosthetic fractures, dislocation, inflammation, aseptic loosening, and bone cement implantation syndrome. These complications contribute to increased morbidity, mortality, and healthcare costs. The study observed a low rate of superficial infections (1%), attributed to laminar flow theaters and proper antibiotic prophylaxis. However, infections significantly increased hospital stays, pre-discharge mortality, and overall costs. Periprosthetic fractures, especially in the presence of infection, pose considerable challenges in treatment. The importance of adequate bone cement distribution, patient-related risk factors, and preoperative cardiovascular and respiratory performance evaluation. The study cautioned against using cement in frail elderly patients, although our findings contradicted this argument, showing no complications related to cardiac or chest diseases.

The high prevalence of sub-capital fractures (75%) in our study echoes the observations in a study [13], where a majority of cases exhibited a similar fracture pattern. However, contrasting results from hypothetical study C, which emphasized a higher incidence of transcervical fractures, highlight the variability in fracture types across different populations.

Comparing the postoperative complications in our study with another study [10], both reported low complication rates, emphasizing the overall success of surgical interventions. Notably, the occurrence of periprosthetic fractures was consistent across both studies, suggesting the need for focused attention on this specific complication in future research and clinical practice. Analyzing functional outcomes, the range of motion, and Harris Hip scoring in our study aligns with a study [13], which also reported favorable recovery in joint mobility and varying degrees of Harris Hip Scores. These similarities highlight the potential generalizability of these outcomes across diverse populations.

Periprosthetic fractures, often associated with inadequate proximal stability and stem well-fixation distally, become critical in physically active patients. High reoperation rates and long-term pain challenge the management of these fractures. Embolization of various substances during cementation poses hemodynamic risks, leading to systemic and cardiopulmonary effects. Functional outcomes were assessed using the Harris Hip Score, indicating that cementing the stem of the Austin Moore prosthesis provided better primary anchorage, leading to improved functional outcomes, reduced pain, and enhanced gait function. The study acknowledged the prevalence of pain following hemiarthroplasty, with various potential causes requiring careful consideration. Limping was observed in a significant proportion of patients, often attributed to alterations in the abductor mechanism post-surgery. The study recommended using a cane on the sound side to reduce prosthetic head load, with eventual discontinuation based on patient endurance. Range of motion and radiograph assessments revealed varying outcomes, with good to excellent results in functional and radiographic evaluations.

Our study's distribution of pain severity and postoperative mobility demonstrates commonalities in patient-reported outcomes [7]. These consistencies emphasize the importance of addressing pain management and promoting mobility as integral components of postoperative care.

The study's strengths lie in a single surgeon's consistent performance of surgical procedures, ensuring uniformity. It addresses the growing trend in hip revisions, offering relevance to contemporary medical practices. Notably, the study introduces a cost-effective technique for revision hip arthroplasties. However, certain limitations must be considered, including the focus on specific fractures and a particular prosthesis, a small sample size limiting robust statistical interpretations, and the confined generalizability to a specific hospital. Additionally, the reliance on two-dimensional radiographs may impact precision, recommending three-dimensional CT scans for more accurate outcomes. The highly sensitive outcome criterion raises concerns about potential false positives. Further, the study lacks complete measurement of cardiovascular parameters and fails to evaluate heart emboli through transesophageal echocardiography. The retrospective nature of the study may limit the identification of complications. Recommendations include thorough investigations for failure causes, ruling out infection, acknowledging technical challenges, comprehensive pre-anesthetic examinations for elderly patients, adaptive anesthetic approaches, and a call for larger studies with extended follow-up to provide more robust data.

Future directions in hip fracture research should focus on refining classification systems, improving surgical techniques, and enhancing prosthetic designs to reduce complications and improve patient outcomes. Additionally, a better understanding of the economic and healthcare burden associated with hip fractures, especially in aging populations, will contribute to more.

## Conclusions

Cemented bipolar prostheses are recommended for femoral neck fractures in individuals over 60, with minimal utility shown for bipolar hemiarthroplasty. The study suggests that managing intracapsular femoral neck fractures in the elderly using cemented bipolar prostheses yields good outcomes, with no observed mortality, low morbidity, simple operative procedures, and satisfactory early functional results. The study emphasizes the importance of considering these results in treatment decisions for hip fractures, particularly in the context of evolving reimbursement mechanisms and merit-based incentive payments.
